# Suffruyabiosides A and B, Two New Monoterpene Diglycosides from Moutan Cortex

**DOI:** 10.3390/molecules17054915

**Published:** 2012-04-30

**Authors:** Rie Furuya, Honghai Hu, Zhenya Zhang, Hideyuki Shigemori

**Affiliations:** Graduate School of Life and Environmental Sciences, University of Tsukuba, Tsukuba, Ibaraki, 305-8572, Japan

**Keywords:** monoterpene diglycoside, suffruyabiosides A and B, human lung adenocarcinoma epitherial A549 cells, radical scavenging effect

## Abstract

Two new monoterpene diglycosides, suffruyabiosides A (**1**) and B (**2**), and seven known compounds **3**-**9** were isolated from Moutan Cortex (root cortex of *Paeonia suffruticosa *Andrews). The structures were elucidated on the basis of 2D NMR spectral data. Suffruyabiosides A (**1**) and B (**2**) are rare monoterpene diglycosides, including a cellobiose in the molecules. Salicylpaeoniflorin (**4**) had a antiproliferation effect similar to paeoniflorin (**3**) on human lung adenocarcinoma epitherial A549 cells. Galloylpaeoniflorin (**8**) and salicylpaeoniflorin (**4**) revealed a more pronounced radical scavenging effect than α-tocopherol (positive control). An increase in the number of phenolic hydroxyl groups produced a more effective radical scavenging effect [**8** > mudanpioside E (**6**) > oxypaeoniflorin (**5**)]. Comparison of the effects of **4** and **5** showed that *o*-substitution with a phenolic hydroxyl group was more effective than *p*-substitution. The results suggest that salicylpaeoniflorin (**4**) may be useful as a cytotoxic and a radical scavenging agent.

## 1. Introduction

Moutan Cortex (root cortex of *Paeonia suffruticosa* Andrews) is one of the most important natural medicines in China, Korea, and Japan. The main production places of Moutan Cortex are Anhui, Sichuan, Gansu, Shaanxi, Hubei, Hunan, Shandong, Guizhou and other places in China. It is clinically used for its analgesic and anti-inflammatory properties, as well as a remedy for female genital diseases [1,2,3,4]. Moreover, the monoterpene glycosides isolated from Moutan Cortex are known for their radical scavenging effect [5]. In a recent study, paeoniflorin (**3**) exhibited antiproliferative activity against human lung adenocarcinoma epithelial A549 cells by arresting cell cycle progression in the G_0_/G_1_ phase and inducing apotosis [6]. However, the structure-activity relationships of the related monoterpene glycosides, including paeoniflorin (**3**), have not been studied in A549 cells. In this study, we isolated two new monoterpene diglycosides, suffruyabiosides A (**1**) and B (**2**), and known compounds **3**–**9** from Moutan Cortex. In addition, we examined the structure-activity relationships of cytotoxic activity on A549 cell and radical scavenging effect of compounds **1**–**9**.

## 2. Results and Discussion

The EtOH extract of Moutan Cortex (1.0 kg) was partitioned between EtOAc and H_2_O, and then the H_2_O-layer was partitioned with *n*-BuOH. The *n*-BuOH layer was separated by column chromatography on silica gel and ODS, and using HPLC to give two new monoterpene diglycosides, suffruyabiosides A (**1**, 0.00028%) and B (**2**, 0.00013%), together with seven known compounds **3**–**9**. The known compounds **3**–**9** were identified as paeoniflorin (**3**) [3], salicylpaeoniflorin (**4**) [7], oxypaeoniflorin (**5**) [8,9], mudanpioside E (**6**) [10], mudanpioside D (**7**) [10], galloylpaeoniflorin (**8**) [2,9], and mudanpioside I (**9**) [11].

**Figure 1:**
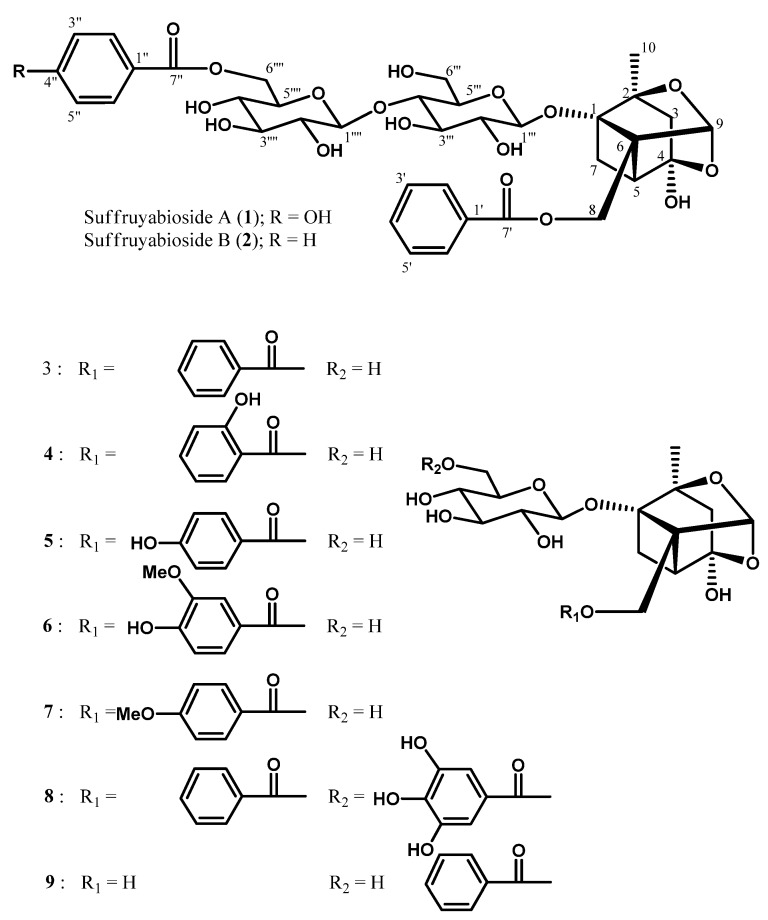
Structures of suffuruyabiosidesA (**1**) and B (**2**), and seven known compounds **3**–**9**.

**Figure 2:**
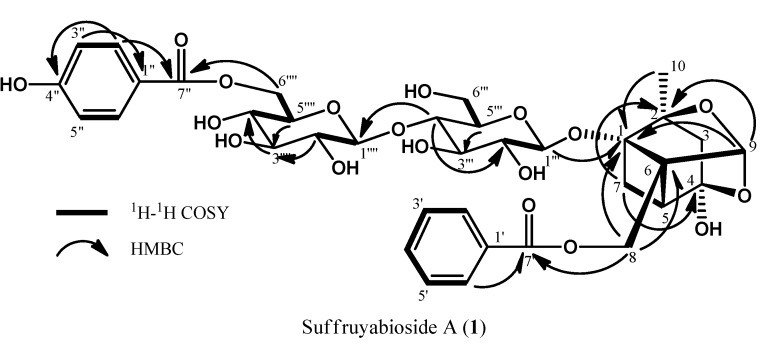
2D NMR correlations of suffuruyabioside A (**1**).

The molecular formula of suffruyabioside A (**1**) was assigned as C_36_H_42_O_18_ by HRESIMS [*m/z* 785.2212 (M+Na)^+^, Δ −5.7 mmu]. The IR spectrum indicated the presence of hydroxyl (3412 cm^−1^) and ester (1729 cm^−1^) moieties. The ^1^H- and ^13^C-NMR spectra of **1** (Table 1) revealed signals assignable to a monoterpene pinane type skeleton similar to that of paeoniflorin (**3**) [12] with signals for three methylenes at δ_H_ 2.21 (1H, d, *J* = 13.0 Hz, H-3), 1.73 (1H, d, *J* = 13.0 Hz, H-3), 1.73 (1H, brd, *J* = 12.3 Hz, H-7), 2.50 (1H, m, H-7), and 4.87 (2H, s, H-8), two methines at δ_H_ 2.52 (1H, m, H-5) and 5.40 (1H, s, H-9), and one methyl at δ_H_ 1.25 (3H, s, H-10). HMBC correlations (Table 1, Figure 1 and Figure 2) of H-5, H-7, H-8, H-9, and H-10 to C-1 (δ_C_ 90.1), H-7, H-9, and H-10 to C-2 (δ_C_ 88.8), H-10 to C-3 (δ_C_ 44.7), H-3, H-5, and H-7 to C-4 (δ_C_ 106.1), H-3 to C-5 (δ_C_ 41.1), H-8 to C-6 (δ_C_ 72.5), H-5 to C-7 (δ_C_ 27.1), and H-8 to C-9 (δ_C_ 103.0) revealed the presence of a pinane type skeleton. The ^1^H-^1^H COSY connectivities of H-1"' to H-2"', H-5"' to H-6"', H-1"" to H-2"", and H-4"" to H-6"" and HMBC correlations of H-2"' to C-4"' (δ_C_ 82.1), H-4"' to C-2"' (δ_C_ 75.4), H-5"' to C-3"' (δ_C_ 78.7), H-1"", H-4"", and H-5"" to C-3"" (δ_C_ 72.4), and H-2"" and H-6"" to C-4"" (δ_C_ 74.7) implied the presence of two sugar moieties. NOESY correlations of H-1"' to H-3"', H-1"' to H-5"', H-1"" to H-3"", H-1"" to H-5"", and H-2"" to H-4"" and two anomeric proton signals of H-1"' and H-1"" observed a doublet at δ_H_ 4.64 (1H, *J* = 7.8 Hz) and 4.44 (1H, *J* = 7.8 Hz), suggesting the presence of two β-glucose moieties. Moreover the presence of benzoyl and *p*-hydroxybenzoyl groups was revealed by the ^1^H-^1^H COSY connectivities of H-2' to H-6', H-2" to H-3", and H-5" to H-6" and HMBC correlations of H-2' and H-6' to C-4' (δ_C_ 135.3) and C-7' (δ_C_ 168.3), H-3" and H-5" to C-1" (δ_C_ 120.0), and H-2" and H-6" to C-7" (δ_C_ 168.8) and C-4" (δ_C_ 163.6). HMBC correlations of H-8 (δ_H_ 4.87) to C-7', H-4"' (δ_H_ 3.64) to C-1"" (δ_C_ 105.7), and H-6""(δ_H_ 4.86) to C-7" (δ_C_ 168.8) revealed that the benzoyl group was attached to C-8 (δ_C_ 63.8) by the ester linkage, the first sugar was linked to C-1"' (δ_C_ 100.6) with the other sugar attached to C-4"' (δ_C_ 82.1) by ether linkages, and the *p*-hydroxybenzoyl group was connected to C-6"" (δ_C_ 65.4) of the other sugar through an ester linkage. 

**Table 1:** ^13^C- and ^1^H-NMR data of suffruyabiosides A (**1**) and B (**2**) in CD_3_OD.

	Suffruyabioside A (1)				Suffruyabioside B (2)		
	δ_C_	δ_H_, *J* in Hz	HMBC *^a^*		δ_C_	δ_H_, *J* in Hz	HMBC *^a^*
1	90.1, C	-			90.1, C	-	
2	88.8, C	-			87.8, C	-	
3	44.7, CH_2_	1.73, d, 13.0	4, 5		41.4, CH_2_	1.76, d, 13.9	2, 5
		2.21, d, 13.0				2.18, d, 13.9	
4	106.1, C	-			104.0, C	-	
5	41.1, CH	2.52, m	1, 4, 7		40.7, CH	2.56, m	3, 7
6	72.5, C	-			72.5, C	-	
7	27.1, CH_2_	1.73, bd, 12.3	1, 2, 4		24.2, CH_2_	1.67, d, 9.5	1, 2, 5
		2.50, m				2.56, m	
8	63.8, CH_2_	4.87, s	1, 6, 9, 7'		62.8, CH_2_	4.80, s	9, 7'
9	103.0, CH	5.40, s	1, 2		102.9, CH	5.50, s	2
10	22.5, CH_3_	1.25, s	1, 2, 3		20.4, CH_3_	1.22, s	1, 2, 3
1'	131.1, C	-			132.1, C	-	
2'	130.7, CH	8.08, dt, 7.8, 1.2	3', 4', 6', 7'		131.5, CH	8.09, dt, 7.6, 1.4	1', 3', 4', 7'
3'	130.6, CH	7.53, t, 7.8	1', 2', 5'		130.6, CH	7.54, t, 7.6	2', 4'
4'	135.3, CH	7.64, tt, 7.4, 1.2	2', 3', 5', 6'		135.3, CH	7.66, tt, 7.6, 1.4	3', 5'
5'	130.6, CH	7.53, t, 7.8	1', 2', 3'		130.6, CH	7.54, t, 7.6	4', 6'
6'	130.7, CH	8.08, dt, 7.8, 1.2	2', 3', 4', 7'		131.5, CH	8.09, dt, 7.6, 1.4	1', 4', 5', 7'
7'	168.3, C	-			168.3, C	-	
1"	120.0,C	-			132.0,C	-	
2"	133.8, CH	7.93, dd, 8.8, 1.9	4", 6", 7"		131.4, CH	8.07, dt, 8.3, 1.3	1", 3", 4", 7"
3"	112.0, CH	6.86, dd, 8.8, 1.9	1", 5"		130.4, CH	7.51, t, 8.3	2", 4"
4"	163.6, C	-			135.2, CH	7.65, tt, 8.3, 1.3	3", 5"
5"	112.0, CH	6.86, dd, 8.8, 1.9	1", 3"		130.4, CH	7.51, t, 8.3	4", 6"
6"	133.8, CH	7.93, dd, 8.8, 1.9	2", 4", 7"		131.4, CH	8.07, dt, 8.3, 1.3	1", 4", 5", 7"
7"	168.8, C	-			168.8, C	-	
1"'	100.6, CH	4.64, d, 7.8	1, 5"'		100.6, CH	4.65, d, 7.8	1, 5"'
2"'	75.4, CH	3.34, m	4"'		75.5, CH	3.35, m	4"'
3"'	78.7, CH	3.35, m	1"', 5"'		79.1, CH	3.30, m	1"', 5"'
4"'	82.1, CH	3.64, m	2"', 1""		82.2, CH	3.64, m	2"', 1""
5"'	77.1, CH	3.39, m	3"'		77.1, CH	3.39, m	3"'
6"'	63.3, CH_2_	3.69, dd, 11.9, 5.7	5"'		63.3, CH_2_	3.70, dd, 12.0, 6.3	5"'
		3.93, dd, 11.9, 2.0	5"'			3.93, dd, 12.0, 2.0	4"'
1""	105.7, CH	4.44, d, 7.8	3""		105.7, CH	4.44, d, 7.8	3""
2""	75.6, CH	3.38, m	1"", 4""		75.6, CH	3.40, m	4""
3""	72.4, CH	3.33, m	1"", 5""		72.8, CH	3.30, m	1"", 5""
4""	74.7, CH	3.68, m	2"", 3""		74.7, CH	3.60, m	2"", 3""
5""	78.7, CH	3.86, m	3""		78.7, CH	3.81, m	3""
6""	65.4, CH_2_	4.65, dd, 12.8, 5.1			65.4, CH_2_	4.66, dd, 11.9, 3.8	7", 4""
		4.86, dd, 12.8, 2.0	7", 4""			4.86, dd, 11.9, 2.0	7"

_a_ HMBC correlations are from carbon stated to the indicated proton(s).

The stereochemistry at various stereocenters of the monoterpene unit was assigned on the basis of similarity of the reported spectral data [13]. On the basis of these observations **1 **was established as 6""-*O*-*p*-hydroxybenzoyl-6"'-*O*-β-glucopyranosyl- paeoniflorin.

The ^1^H and ^13^C-NMR spectra (Table 1) of suffruyabioside B (**2**) also resembled that of paeoniflorin (**3**), except for the additional appearance of sugar and benzoyl proton signals. The molecular formula was established as C_36_H_42_O_17_ by HRESIMS data [*m/z* 769.2281 (M+Na)^+^, Δ−3.9 mmu]. Analysis of the IR spectrum of **2** suggested the presence of hydroxyl (3388 cm^−1^) and ester (1711 cm^−1^) groups in the molecule. The ^1^H and ^13^C-NMR data indicated the presence of the same monoterpene pinane type skeleton as in **3** (Table 1). The two sugar NMR signals at H-1"' (δ_H_ 4.65, *J *= 7.8 Hz; δ_C_ 100.6) and H-1"" (δ_H_ 4.44, *J *= 7.8 Hz; δ_C_ 105.7) and NOESY correlations of H-1"' to H-3"', H-1"' to H-5"', H-1"" to H-3"", and H-1"" to H-5"" revealed the presence of two β-glucose moieties. Furthermore, analysis of the HMBC spectrum (Table 1) clearly indicated that the anomeric carbon C-1"" (δ_C_ 105.7) was coupled with H-4"' (δ_H_ 3.64) through the ether linkage. HMBC correlations of H_2_-8 (δ_H_ 4.80) to C-7' (δ_C_ 168.3) indicated that the benzoyl group was attached to C-8 (δ_C_ 62.8) of the aglycone by the ester linkage, while the other benzoyl group was connected to C-6"" (δ_C_ 65.4) of Glc2 by the ester linkage on the basis of HMBC correlation of H-6"" (δ_H_ 4.66 and 4.86) to C-7" (δ_C_ 168.8). Therefore, **2 **was identified as 6""-*O*-benzoyl-6"' -*O*-β-glucopyranosylpaeoniflorin.

**Figure 3:**
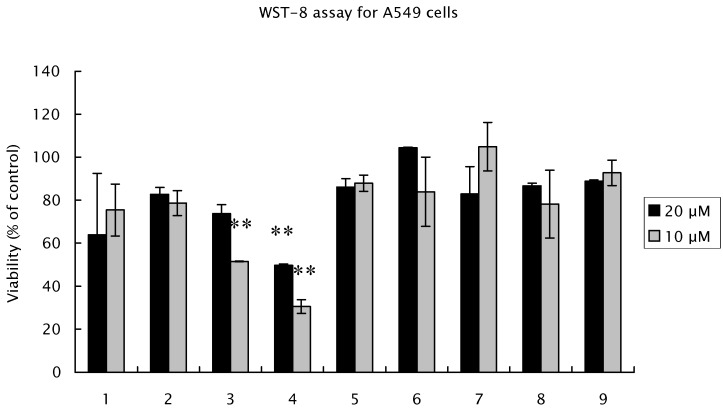
Antiproliferative effects of suffruyabiosidesA (**1**) and B (**2**), and known compounds **3**-**9** for A549 cells. Cells were incubated with different concentration of each compound for 48 h in 96-well plates. Proliferation was measured by WST-8 Assay. Results are expressed as percentage cell proliferation relative to the control group. This assay was repeated two times. Data are the mean ± SD of three determinations. ** significant difference (*p* < 0.01) *vs*. control cells. (Dunnett’s test).

The cytotoxic activities of suffruyabiosides A (**1**) and B (**2**) and known compounds **3**-**9** against human lung adenocarcinoma epithelial A549 cells were assayed (Figure 3). Salicylpaeoniflorin (**4**) and paeoniflorin (**3**) showed moderate cytotoxicities (viabilities: 31% and 52%, respectively), while other compounds including suffruyabiosides A (**1**) and B (**2**), showed very weak activities (viabilities: 63–100%).

**Figure 4:**
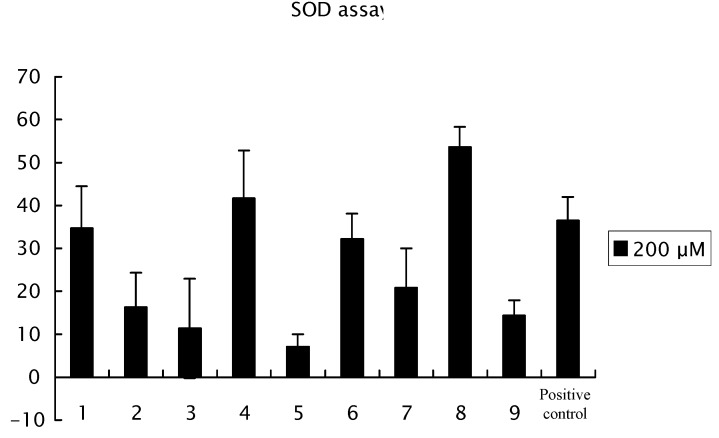
Radical scavenging effects of suffruyabiosides A (**1**) and B (**2**) and known compounds **3**–**9**. After the incubation of the plate at 37 °C for 20 min, read the absorbance at 450 nm using an ELISA reader. This assay was repeated two times. Data are the mean ± SD of three determinations.

The structure-activity relationships for radical scavenging with isolated compounds **1**–**9** are shown in Figure 4. Galloylpaeoniflorin (**8**) was the most effective among the nine compounds **1**–**9**. Galloylpaeoniflorin (**8**) and salicylpaeoniflorin (**4**) revealed a more pronounced radical scavenging effect than α-tocopherol (positive control). Increase in the number of phenolic hydroxyl groups produced a more effective of radical scavenging effect (**8** > **6** > **5**). Comparing the effect of **4** with **5** showed that *o*-substitution with a phenolic hydroxyl group was more effective than *p*-substitution. In addition, mudanpioside I (**9**) was more effective than paeoniflorin (**3**), indicating that a benzoyl group connected to a C-6 of the β-glucose by the ester linkage was more effective for radical scavenging than a benzoyl group attached to the C-8 monoterpene pinane type skeleton.

In conclusion, two new monoterpene glycosides, suffruyabiosides A (**1**) and B (**2**) were isolated from Moutan Cortex. It was the first reported as having a cellobiose in paeoniflorin related compounds [9]. Salicylpaeoniflorin (**4**) exhibited both cytotoxic activity against A549 cells and a radical scavenging effect in an SOD assay, suggesting that salicylpaeoniflorin (**4**) may be useful as a cytotoxic and a radical scavenging agent.

## 3. Experimental

### 3.1. General

Optical rotations were measured with a JASCO DIP-370 polarimeter. IR spectra were recorded on a JASCO FT/IR-300 spectrometer. ^1^H-NMR (500 MHz) and ^13^C-NMR (125 MHz) spectra were measured and recorded in CD_3_OD on a Bruker Avance 500 spectrometer. Chemical shift values (δ) were reported in parts per million (ppm) relative to CD_3_OD (δ_H_ 3.35, δ_C_ 49.8). HRESIMS were recorded on Waters Xevo Q-Tof. 

### 3.2. Plant Material

Moutan Cortex (the roots cortex of *Paeonia suffruticosa* Andrews.) was purchased from Beijing Tong Ren Tang Group Co., Ltd. in November 2007, which was collected from Anhui Province in China and dried by sunlight. This sample was paeoniaceae plant peony root bark, and the root was removed the wooden heart, washed, sliced, and dried. A voucher specimen has been deposited at the Graduate School of Life and Environmental Sciences, University of Tsukuba, Tsukuba, Japan.

### 3.3. Extraction and Isolation

Moutan Cortex (1.0 kg) was crushed with homogenizer and extracted three times with EtOH (2L × 3) overnight at room temperature, followed by removal of solvent under reduced pressure, to yield a dried EtOH extract (340 g). The EtOH extract was dissolved in H_2_O (900 mL) and extracted with EtOAc (900 mL × 3). The H_2_O-soluble portion was partitioned between *n*-BuOH (900 mL × 3) and aqueous layers. The *n*-BuOH layer (16.4 g) was chromatographed on a silica gel using CHCl_3_-MeOH-H_2_O (65:25:4 → 5:5:1) to afford MC-BU-1~11. Further MC-BU-4 was separated using chromatograph on an ODS with MeOH-H_2_O (1:9 → 3:7 → 1:1), to give 8 fractions MC-BU-4-1~8. MC-BU-4-4 was subjected to passage over an ODS HPLC column (TSK-gel ODS-80Ts, TOSOH, ϕ 7.8 × 300 mm, MeOH-H_2_O = 20:80 → 100:0, 1.0 mL/min) to give oxypaeoniflorin (**5**, 25.3 mg). MC-BU-4-4-12 was further purified using an ODS HPLC column (TSK-gel ODS-80Ts, TOSOH, ϕ 4.6 × 250 mm, MeOH: H_2_O = 20:80 → 100:0, 1.0 mL/min) to give mudanpioside E (**6**, 1.4 mg). MC-BU-4-5 (548.5 mg) was subjected to passage over an ODS HPLC column (TSK-gel ODS-80Ts, TOSOH, ϕ 7.8 × 300 mm, MeOH-H_2_O = 20:80 → 100:0, 1.0 mL/min) to give paeoniflorin (**3**, 127.6 mg) and mudanpioside D (**7**, 0.9 mg). MC-BU-4-5-8 was further purified by an ODS HPLC column (TSK-gel ODS-80Ts, TOSOH, ϕ 7.8 × 300 mm, MeOH-H_2_O = 30:80 → 100:0, 1.0 mL/min) to give salicylpaeoniflorin (**4**, 2.6 mg). MC-BU-4-6 (79.2 mg) subjected to passage over an ODS HPLC column (TSK-gel ODS-80Ts, TOSOH, ϕ 7.8 × 300 mm, MeOH-H_2_O = 35:65 → 100:0, 1.0 mL/min) to give 9 fractions MC-BU-4-6-1~9. MC-BU-4-6-6 and MC-BU-4-6-7 were purified to give galloylpaeoniflorin (**8**, 4.7 mg) and mudanpioside I (**9**, 3.3 mg), respectively. MC-BU-4-6-8 was then purified by separation over an ODS HPLC column (TSK-gel ODS-80Ts, TOSOH, ϕ 4.6 × 250 mm, MeOH-H_2_O = 40:60 → 100:0, 0.8 mL/min) to give suffruyabioside A (**1**, 2.8 mg). MC-BU-4-6-9 further purified using an ODS HPLC column (TSK-gel ODS-80Ts, TOSOH, ϕ 4.6 × 250 mm, MeOH-H_2_O = 50:50 → 100:0, 1.0 mL/min) to give suffruyabioside B (**2**, 1.3 mg).

*Suffruyabioside A *(**1**). Colorless amorphous solid; [α]_D_^27^ −18.0 (*c *= 1.00, MeOH); IR (KBr) ν_max_ 3412, 1729, 1525, 1404, and 1069 cm^−1^; UV (MeOH) λ_max_ nm (ε) 206 (26500), 226 (18400), and 258 (13700); ^1^H and ^13^C-NMR see Table 1; ESIMS *m/z* 785 [M+Na]^+^; HRESIMS *m/z* 785.2212 [M+Na]^+^, calcd for C_36_H_42_O_18_Na, 785.2269. 

*Suffruyabioside B * (**2**). Pale yellow amorphous solid; [α]_D_^28^ −14.0 (*c *= 1.00, MeOH); IR (KBr) ν_max_ 3388, 1711, 1459, 1357, and 1078 cm^−1^; UV(MeOH) λ_max_ nm (ε) 202 (18700), 226 (26300), and 258 (4500); ^1^H and ^13^C-NMR: see Table 1; ESIMS *m/z* 769 [M+Na]^+^; HRESIMS *m/z *769.2281 [M+Na]^+^, calcd for C_36_H_42_O_17_Na, 769.2320.

### 3.4. Preparation of Paeoniflorin and Other Compounds

Suffruyabiosides A (**1**) and B (**2**) and other seven known compounds **3**–**9** were dissolved in water and stored at −30 °C. For all cell proliferation assay, the final concentrations of the test compounds were prepared by diluting the stock with RPMI-1640. Control cultures also received the carrier solvent RPMI-1640.

### 3.5. Cell Culture

Human lung adenocarcinoma epithelial A549 cells (ATCC CCL 185, RIKEN Cell Bank) was maintained in monolayer culture at 37 °C and 5% CO_2_ in RPMI-1640 supplemented with 10% FBS, 100 units/mL penicillin G and 100 µg/mL streptomycin.

### 3.6. Cell Proliferation Assay

Inhibition of cell proliferation for A549 cell by suffruyabiosides A (**1**) and B (**2**) and other seven known compounds **3**–**9** was measured using the WST-8 assay. Briefly, cells were placed in 96-well culture plates (1 × 10^4^ cells/well). After 24 h incubation, cells were then incubated for 48 h in the presence or absence of compounds **1**–**9** (20 and 10 µM) dissolved in culture medium and an appropriate solvent. The WST-8 test solution (10 µL) was added to each well. After 4 h incubation, absorbance was measured on ELISA reader (Microplate Reader Model 550, Bio-RAD) at the test wavelength of 450 nm. RPMI-1640 supplemented with 10% FBS was used as the negative control.

### 3.7. Radical Scavenging Effect with SOD

Suffruyabiosides A (**1**) and B (**2**) and other seven known compounds **3**–**9** were subjected to a superoxide dismutase (SOD) assay using the SOD Assay Kit-WST according to the technical manual. Briefly, in a 96-well plate, 20 µL of sample solution was added to each sample and blank 2 well, and 20 µL of double distilled water was added to each blank 1 and blank 3 well. Then 200 µL of WST working solution was added to each well. After mixing, 20 µL of dilution buffer was added to each blank 2 and blank 3 well, and 20 µL of enzyme working solution (15 µL of enzyme mixted with 2.5 µL dilution buffer) was added to each sample and blank 1 well. The plate was incubated at 37 °C for 20 min and the O.D. was determined at 450 nm using an ELISA reader (Microplate Reader Model 550, Bio-RAD). The SOD-like activity was calculated by the following equation:

SOD activity (inhibition rate %) = {[(A blank 1–A blank 3)–(A sample–A blank 2)] / (A blank 1–A blank 3)}× 100

where A blank 1, A blank 2, A blank 3, and A sample were the absorbance of blank 1, blank 2, blank 3, and the sample, respectively.

## 4. Conclusions

Two new monoterpene diglycosides, suffruyabiosides A (**1**) and B (**2**), and seven known compounds **3**–**9** were isolated from Moutan Cortex (root cortex of *Paeonia suffruticosa *Andrews). Suffruyabiosides A (**1**) and B (**2**) are rare monoterpene diglycosides, including a cellobiose in the molecules. Salicylpaeoniflorin (**4**) had a cytotoxic effect on human lung adenocarcinoma epitherial A549 cells and radical scavenging effect.
